# Structure and Function of Membrane Proteins

**DOI:** 10.3390/ijms24098350

**Published:** 2023-05-06

**Authors:** Larry Fliegel

**Affiliations:** Department of Biochemistry, University Alberta, Edmonton, AB T6G 2H7, Canada; lfliegel@ualberta.ca; Tel.: +1-780-492-1848

While we have a great deal of information on the human genome, in many cases we still know little about the structure’s function, the regulation of membrane proteins and how they are altered in health and disease. Genome sequencing projects show that 20 to 30% of open reading frames encode membrane proteins and in higher organisms the fraction of membrane proteins is elevated. In humans, up to 43% of all proteins are embedded in or cross a membrane [1]. It is also estimated that more than 50% of drugs marketed target integral membrane proteins, including G-protein coupled receptors, ion channels and solute carrier transporters [2]. The involvement of membrane proteins in disease is unquestionable and includes relatively common diseases such as muscular dystrophy [3] and cystic fibrosis [4]. Until relatively recently, only a small fraction of the proteins that have been analyzed in any detail or had their structure elucidated are membrane proteins. Recent advances in technology, including cryo-electron microscopy, protein production and other techniques, have greatly improved our understanding of membrane proteins [5,6]. They have led to new insights into the membrane protein structure and allow more direct testing of hypotheses based on a firmer knowledge of the protein. Additionally, it has been possible to use this newly gained knowledge to benefit other studies on different related membrane proteins. This Special Issue of the *International Journal of Molecular Sciences* provides nine examples of studies on the structure and function of membrane proteins, including reviews and original articles. These are summarized below.

The first paper Is that of the Dibrov group [7] and is the study of the cation proton antiporter of *Vibrio cholerae* NhaP type. The protein is critical in the acid tolerance response of *V. cholerae*, allowing the cells to survive in acidic environments and especially in K^+^-rich environments. The NhaP2 isoform is the isoform of most importance of the known trio. The paper uses information from silico structure modeling, molecular dynamics simulations and extensive mutagenesis studies to propose that the ion-motive-binding module of VC-NhaP2 is made of two functionally different regions. One region is a suggested cation binding pocket which is formed from antiparallel unfolded parts of transmembrane segments five and twelve that cross each other mid membrane. The other is a specific cluster of amino acids that determine ion selectivity. The authors also discuss the interesting hypothesis of “ligand shading” in the active center of Vc-NhaP2, whereby alkali cations use overlapping but not identical sets of ligands, thus differently affecting protonation of the antiporter during the catalytic cycle. Vc-NhaP2 is a potential target for antimicrobials that target this pathogen and the results of studies on the function of this antiporter are important for the development of inhibitors of this protein [7]. 

The second paper in the Special Issue is a review of the Erk1/2-induced structural and functional changes in the Na^+^/H^+^ exchanger isoform 1 [8]. The human Na^+^/H^+^ exchanger isoform 1 (NHE1) is a ubiquitous plasma membrane protein that removes one intracellular H^+^ in exchange for one extracellular sodium. It thereby regulates the intracellular pH. It is also important clinically in heart disease and in cancer cell migration, invasion, and metastasis. NHE1 has a 500 amino acid membrane domain responsible for transport and a 315 amino acid cytosolic regulatory domain with structured and unstructured regions. Erk1/2 are activated by many stimuli, including hormonal activation and activation in disease states such as ischemia reperfusion. The protein kinase ERK binds to the NHE1 tail and Erk1/2 phosphorylate NHE1 at multiple locations. This enhances NHE1 activity, with subsequent downstream effects on cells and tissues. The phosphorylation also stabilizes disordered regions of the regulatory tail. This type of regulation, involving the change of an intrinsically disordered region to an ordered domain, is a newly characterized important method of protein regulation in cellular signaling [8,9]. How the change in conformation of the NHE1 regulatory tail causes an alteration in activity of the membrane domain has yet to be elucidated. 

Another article in this series is a review on the biophysical methods of characterization of membrane proteins by the Moraes group [2]. As stated in the publication, integral membrane proteins are very challenging to study, often due to the low levels of expression and instability once removed from their native membrane. Additionally, though membrane proteins are important drug targets, many drugs have unwanted side effects or a lower than desired efficacy, which may be due to poor understanding of the target protein. The review explains many useful methods that assist greatly in analysis and screening for the optimal conditions for membrane proteins. These will be briefly summarized. Dynamic light scattering is a simple and powerful method to characterize particles in solution. Particles are illuminated with laser light and their Brownian motion coupled with their size gives specific information on their dimensions. The procedure analyzes protein behavior in solution, particularly information on aggregation and stability. It can also give information on protein behavior in the crystallization process. Another technique is characterization of membrane proteins using size exclusion chromatography with multi-angle light scattering (SEC-MALS). SEC-MALS can determine the oligomeric behavior of membrane proteins in solution. The technique separates proteins by size and can determine their composition and mass. Circular dichroism (CD) can be used to study the conformational behavior of proteins in solution in varying environments, including ionic strength, membrane mimics and ligand binding interactions. CD can rapidly detail the secondary structure content of proteins, including the number and length of α-helices and ℬ-strands. CD is related to the dichroism of polarized light, with differential absorption of left- and right-handed light. Synchrotron radiation circular dichroism enables small aperture, long pathlength cuvette cells to be used. Fluorescence-dye-based differential scanning fluorimetry (DSF) is a tool that can determine the membrane protein stability in the presence of small molecules or ligands in the presence of different buffers or detergents. It can be used to screen buffers and ligands that stabilize a protein and can assess the effects of mutations. Mid-infrared spectroscopy is a simple procedure to measure the concentration of proteins, although it has a low measurement range. It is much less dependent on amino acid composition than absorption at 280 nm. Lipid cubic phase (LCP) recovery after photobleaching can serve to assist in identifying the best lipid/protein constructs that may not promote diffusion in LCP. It can be used to screen for better protein constructs, ligands, better lipids and additives. The idea is to improve crystallization trials, increasing the chance of finding the right crystallization conditions. The authors provide details on all these biophysical characterization methods, and the pros and cons and usefulness of each technique. They provide an excellent summary of these features in Table 1 of their paper.

An article in this Special Issue by the group of Rainey [10] highlights another unique approach to examine membrane proteins, in this case studying ligand–receptor binding at the atomic level. In this unique study, they exploit the lack of natural fluorine in proteins. The ^19^F nucleus is used as it responds to environmental changes, allowing for a one-dimensional NMR study. Their study examined the apelinergic system, which is important in vasodilation, cardiovascular development and insulin homeostasis and is involved in diabetes and chronic heart failure. Their apelinergic system comprises the apelin receptor (AR)/APJ and two types of ligands, apelin and apela peptides. For their experiments, AR fragments were constructed, containing either the N-terminal and first transmembrane α-helix or the first three α-helices. These were prepared with incorporated fluorotryptophan. The labeled AR fragment interactions with the apelin analogue were examined by ^19^FNMR spectroscopy. The binding observed between the two AR fragments and apelin varied, and the shorter construct was not sufficient to recapitulate physiological binding. This technique was not only able to characterize the region needed for binding, but by introducing labels in different locations, they could characterize ligand-specific modulation at defined positions. Probing binding by this technique could even be applied to disease models, examining changes in binding in the disease state. 

Another research article in this Special Issue is by the Dores group [11], who studied activation of the melanocortin-2 receptor. The melanocortin-2 receptor (also known as adrenocorticotropic receptor) is a critical component of the hypothalamic-pituitary-adrenal axis. Their study used the model of the Xenopus melanocortin-2 receptor to study the interaction of the adrenocorticotropic hormone with the receptor. The interactions between the hormone and wild-type and mutant receptors with site-specific alanine mutations were examined in a reconstituted system in CHO cells. The study identified specific residues in *Xenopus tropicalis* melanocortin-2 receptor in transmembrane segments 4 and 5 and extracellular domain 2 that are involved in the activation of the receptor and may be present in the hormone contact site. These novel results were compared to earlier studies on the human melanocortin-2 receptor and the rainbow trout melanocortin-2 receptor to identify common adrenocorticotropic hormone activation features in melanocortin-2 receptors. 

The Cyclin and CBS domain divalent metal cation transport mediators (CNNMs) are a family of four proteins that are involved in magnesium transport across cell membranes. A different research article [12] in the Special Issue examined the structure and ligand binding of the intracellular region of isoform CNNM4, which is predominant in the brain, bone marrow, immune system, and intestinal tract. The CNNM family are proteins that have four independent domains linked by linkers of different lengths. The intracellular region is directly attached by a long α-helix (H0) and comprises two distinct domains, a Bateman module and a cyclic nucleotide monophosphate binding-like domain (cNMP domain). The Bateman module consists of two intertwined cystathionine ℬ-synthase (CBS) motifs that bind ATP and Mg^2+^ ions. This article [12] presents the structures of both the Bateman module and the cNMP domain. Additionally, it derives a model of the entire intracellular region in the presence and absence of ATP and Mg^2+^ ions. They note that the Bateman module interacts with ATP and Mg^2+^ ions at non-overlapping sites, which facilitates cooperativity. Additionally, both domains self-dimerized, contributing to dimer stabilization of the full-length protein. 

A review by the Buchanan group [13] describes the structure and stoichiometry of the Ton complex. The Ton system of Gram-negative bacteria is an inner membrane complex powered by the proton motive force and made of three integral membrane proteins: TonB, ExbB and ExbD. Energy from the proton motive force is propagated through TonB to outer membrane transporters to allow nutrient entry. The review presents the latest findings and recent structures reported. The latest information on how TonB acts as an energy conduit of the system is also presented.

The next article in the series [14] studies the cytosolic regulatory tail of the NHE1 protein. Genetic stop codon polymorphisms of this protein in humans have been found in the C-terminal tail that have aberrant targeting and activity. By progressively shortening the C-terminal regulatory protein and expressing it in live cells, the authors show that amino acids 562–568 are critical in expression and targeting and activity. Site specific mutagenesis confirmed this region’s importance. The results defined a new sequence important in NHE1 activity and protein levels. 

The final article in the series [15] is from the Neuhaus group centered in Kaiserslautern, Germany. It studies the plant salt tolerance protein SOS1. SOS1 is responsible for the movement of intracellular sodium out of plant cells and across the plasma membrane. It has a long regulatory cytosolic tail with an autoinhibitory domain that is active in the absence of phosphorylation by the regulatory SOS2 protein. This study examined regulatory sites for protein–protein interactions of the tail. The C-terminal domain was overexpressed and used as bait for isolated binding proteins. SOS1 bound several previously unknown proteins. This included 14-3-3 proteins that interacted with the tail. Overexpression of the SOS1 C-terminal in plants led to sequestration of inhibitory 14-3-3 proteins and led to more activation of SOS1 and an increased salt tolerance. The results may lead to new biotechnological strategies to improve salt tolerance in agronomically important species to prevent crop failure.

In summary, the series of nine articles are a diverse array of publications on membrane proteins that range from reviews on the structure of membrane proteins and their domains to articles revealing novel information on the structure and regulation of membrane proteins. In this series, we traveled from bacteria proteins, to plant proteins, to human proteins and to *Xenopus* species proteins. This research is important for both the health and welfare of humans and basic science and could have economic and agronomic significance. Several articles describe important techniques used to study membrane proteins and it is hoped that this will benefit future studies by other researchers in the field. 

The future of research on membrane proteins is bright. As noted above, recent advances in cryo-electron microscopy and other techniques have greatly improved our ability to study the structure and function of membrane proteins [5,6]. It is also important to mention that aside from cryo-EM, another new age of understanding of membrane protein structure and function has begun. AlphaFold, a novel approach in which machine learning is used alongside the knowledge of protein structure to predict, with a relatively high level of certainty, the membrane protein structure, has recently been developed [16]. Predictions are based on neural network architecture and on evolutionary, physical and geometric constraints of protein structures. A large number of structures have been deposited and are publicly available. This should certainly facilitate development of inhibitors and a better understanding of protein functions and regulation [17]. Experiments on protein functions can now begin based on the predicted structures, which should facilitate progress in the field. 
